# Use of Tracer Elements for Estimating Community Exposure to Marcellus Shale Development Operations

**DOI:** 10.3390/ijerph17061837

**Published:** 2020-03-12

**Authors:** Maya Nye, Travis Knuckles, Beizhan Yan, James Ross, William Orem, Matthew Varonka, George Thurston, Alexandria Dzomba, Michael McCawley

**Affiliations:** 1 West Virginia University School of Public Health, Department of Occupational and Environmental Health Sciences, Morgantown, WV 26506, USA; mjn0001@mix.wvu.edu (M.N.); tknuckles@hsc.wvu.edu (T.K.); ardzomba@mix.wvu.edu (A.D.); 2 The Lamont-Doherty Earth Observatory at Columbia University, Palisades, NY 10964, USA; yanbz@ldeo.columbia.edu (B.Y.); jross@ldeo.columbia.edu (J.R.); 3 US Geological Survey, Reston, VA 201924, USA; borem@usgs.gov (W.O.); mvaronka@usgs.gov (M.V.); 4 Program in Exposure Assessment and Human Health Effects at the Department of Environmental Medicine, New York University School of Medicine, Tuxedo, NY 10987, USA; George.Thurston@nyumc.org

**Keywords:** tracer elements, unconventional natural gas development, source identification, particulate matter, PM2.5, community exposure

## Abstract

Since 2009, unconventional natural gas development (UNGD) has significantly increased in Appalachia’s Marcellus Shale formation. Elevations of fine particulate matter <2.5 µm (PM2.5), have been documented in areas surrounding drilling operations during well stimulation. Furthermore, many communities are experiencing increased industrial activities and probable UNGD air pollutant exposures. Recent studies have associated UNGD emissions with health effects based on distances from well pads. In this study, PM2.5 filter samples were collected on an active gas well pad in Morgantown, West Virginia, and three locations downwind during hydraulic stimulation. Fine particulate samples were analyzed for major and trace elements. An experimental source identification model was developed to determine which elements appeared to be traceable downwind of the UNGD site and whether these elements corresponded to PM2.5 measurements. Results suggest that 1) magnesium may be useful for detecting the reach of UNGD point source emissions, 2) complex surface topographic and meteorological conditions in the Marcellus Shale region could be modeled and confounding sources discounted, and 3) well pad emissions may be measurable at distances of at least 7 km. If shown to be more widely applicable, future tracer studies could enhance epidemiological studies showing health effects of UNGD-associated emissions at ≥15 km.

## 1. Introduction

Particulate air pollution (PM) is known as a significant contributor to the global burden of disease and mortality. PM is a physicochemically diverse mixture, with a variety of amounts, shapes, sizes and origins—all of which contribute to PM toxicity. PM can be delineated into three sizes PM <10 µm (PM10, coarse), <2.5 µm (PM2.5, fine), and PM <0.1 µm (PM0.1, ultrafine). Smaller-sized particles deposit deep in the lungs, which has been shown to invoke an inflammatory immune response and produce oxidative stress [1,2]. Exposure to mass amounts of each size particle has been linked to adverse health effects, such as adverse birth outcomes [3,4], asthma [5,6,7,8,9,10], cardiovascular disease [9,11,12,13,14] and diabetes [9,15]. In 2015, exposure to PM2.5 was the fifth-ranking mortality risk factor globally, a significant increase from previous years [14,16]. Exposures to short-term changes in PM2.5 from as little as 6.0 [13] to 10 µg/m3 [14,16] have been associated with acute cardiac events [11,12]. Reduced levels of PM2.5 have been linked to improved lung function in vulnerable populations. Reduced PM10 and PM2.5 levels are correlated with reduced respiratory symptoms in children particularly with asthma [17,18]. Therefore, reducing exposure to particulate matter is necessary to support overall population health and reduce health disparities among vulnerable populations.

Since 2009, unconventional natural gas development (UNGD) has significantly increased [19,20]. Elevated concentrations of PM2.5 have been documented in areas surrounding UNGD [21,22,23]. Recent epidemiological studies have associated UNGD emissions with health effects, such as adverse birth outcomes [24,25,26,27], asthma exacerbations [28] and increased cardiologic and neurologic hospitalization in areas around UNGD [29]. These adverse health effects are similar to those associated with particulate matter exposure. According to Thurston et al. (2016), mortality from exposure to combustion from fossil fuel sources is associated with PM2.5 and can vary greatly by source [30].

The scientific literature currently lacks methodologically rigorous studies that link UNGD-related particulate matter with health outcomes [31,32,33]. Other than our own previous work [34], the only studies modelling exposure with collected environmental samples measured volatile organic compounds rather than particulate matter [23,26]. Most studies to date have employed proximity to well as a surrogate of exposure rather than on-the-ground measurements [24,27,28]. What is not known is how much UNGD-derived PM that people are being exposed to, its composition, and at what distances from UNGD operations it persists.

We hypothesized that well pad air emissions could be determined by elemental analysis of collected PM2.5 downwind. To determine how far away from the well pad air emissions can be detected, we tested the hypothesis that tracer elements unique to the UNGD well pad can be identified downwind and off site of the gas well. We also sought to determine whether these tracer elements corresponded to PM2.5 measurements simultaneously collected in multiple stations. In this study, we collected PM2.5 on polytetrafluoroethylene (PTFE) filters at three points downwind of a UNGD well pad in West Virginia during hydraulic fracturing. High-resolution inductively coupled plasma mass spectrometry (HR-ICP-MS) was applied to analyze the filters for major and trace elements (hereinafter, elements or trace elements). Further analysis was conducted using an experimental source identification model incorporating wind patterns to determine which elements, if any, could be traced downwind of the UNGD site.

## 2. Materials and Methods 

### 2.1. Subjects and Materials

No humans or animals were subjected to testing in this study. Permission to conduct environmental sampling was obtained for each site. Researchers obtained International Association of Drilling Contractors (IADC)/SafeLand USA RigPass certification through West Virginia University Safety and Health Extension office, a requirement for conducting sampling on the well pad.

### 2.2. Sampling Locations

In this study, we collected 48 hour samples of PM2.5 at three points predominantly downwind (1, 2 and 7 km) of an active Marcellus Shale gas well pad in Morgantown, West Virginia. Samples were obtained during an 8 day hydraulic fracturing stimulation process that occurred on October 28–November 5, 2015 (Figure S1). Sampling sites were located within the valley of an approximately 100 meter deep river valley in Morgantown, West Virginia (Figure S2) and positioned in relative areas that would benefit from this topography (immediately downhill from the well pad, beside the river, in direct line of sight, and at the end of the river valley). Historic meteorological data were obtained from the Brooks green roof (KWVMORGA25) sampling station in Morgantown, West Virginia accessed by Weather Underground (Weather Underground, 2015). Since these background data indicated that in-valley air flow would primarily occur from south to north, a sampling station set up on the MSEEL gas well pad (Site 0) was established just north of the site, downwind of site activity. Other sampling sites were set up at points approximately 1 km (Site 1), 2 km (Site 2) and 7 km (Site 3) downwind of drilling activity. Site 3 was considered most likely to represent background air quality in the valley due to its distance from the site and previous studies showing 1 km as the expected limit of detectable concentrations from the pad [26]. The selection of these locations was consistent with comparable studies in which atmospheric particulate matter was sampled [35].

Samples were collected every two days at each of the four operational sampling sites (Sites 0, 1, 2, 3) using a Gast Compressor Vacuum Pump DOA-V191-AA (Benton Harbor, MI) during the 8 day stimulation process. Materials were captured on a Teflon Membrane Disc Filter (2 μm, 47 mm, Pall Corporation, Port Washington, NY) utilizing pre-sterilized URG-2000-30FG 47 mm filter holder and the URG-2000-30EH 16.7 Lpm, 2.5 μm Teflon Coated Aluminum Cyclone (URG Corporation, Chapel Hill, NC). Daily flow calibration was conducted using the Sierra 822 Top-Trak Mass Flow Meter (Monterey, CA) and Key Instruments adjustable flowmeter (10-100 SCFH) (Brooks Instrument, Hatfield, PA). Total flow (m3) was tracked utilizing the Kimmon SK25EX (Hi-Q Environmental Products, Azbil Kimmon Co., Ltd, Tokyo, Japan) dry gas meter.

Teflon forceps were used to handle the filters which were then stored in plastic petri dishes and transported immediately from each site in a cooler. Within 60 minutes of collection, samples were transferred for storage at 277.15 K at the West Virginia University Occupational and Environmental Health Sciences laboratory. Samples were shipped overnight on dry ice to the Lamont Doherty Earth Observatory in Palisades, New York, at Columbia University for HR-ICP-MS analysis.

### 2.3. Wind Direction

Wind data were obtained on the Weather Underground online historical database (https://www.wunderground.com/personal-weather-station/dashboard?ID=KWVMORGA25#history/tgraphs/s20151031/e20151031/mdaily last accessed: 8-8-2017) for Morgantown, West Virginia, using the Brooks green roof (KWVMORGA25) sampling station that averaged temperature, humidity, pressure, UV, and wind direction approximately every 9 minutes. Wind data were synchronized with sample collection data using time of the first sample taken at the first site and time of the last sample taken at the last site during the sampling timeframe. For each sampling period, we calculated the percentage of time in which the wind derived from a specific direction, and the speed (<1 mile per hour [mph], 1 to 2 mph, 2 to 5 mph, 5 to 10 mph, >10 mph) at which the wind was blowing. Our primary wind patterns of interest were periods when the wind was blowing greater than 1 mph and derived from Southeast (SE), South–Southeast (SSE), South (S), South–Southwest (SSW), and Southwest (SW). Wind direction data for sampling periods where the wind was not blowing greater than 1 mile per hour and/or in the direction of our sampling stations for more than 5% of the time were excluded.

### 2.4. Elemental Analysis

Elemental analysis of the digested air filters was conducted at the Columbia University Lamont Doherty Earth Observatory using the lab’s Axiom^®^ (VG-Elemental) Thermo Element XR, a magnetic sector high-resolution inductively-coupled plasma mass spectrometer (HR-ICP-MS). The study utilized “Summer New York” analysis methods previously published [36]. This method is frequently employed with low detection limit trace element analyses [35]. Sample preparation included microwave digestion and was amended to include the following: after removing the ring, the filter was placed in a 7 mL vial and combined with 20 µL ethanol, 250 µL HNO3 (optima grade) and 50 µL hydrofluoric acid prior to microwave digestion. Vials were sealed and placed in microwave vessels. A microwave was used to warm samples for 15:00 minutes at 750 W, and rerun at 10:00 minutes at 500 W, 2:30 minutes at 750W, and 10:00 minutes at 500 W. Samples were cooled and the entire cycle was rerun without opening or adding additional reagents. Additionally, three runs of National Institute of Standards and Technology (NIST) standard reference material SRM-1648a (airborne particulate matter) were used to demonstrate precision and accuracy (93% mean recovery). Quantification limits were calculated by taking the standard deviation of the filter blanks and multiplying it by 3 [36]. Results are listed in Table S1.

### 2.5. Experimental Design and Statistical Analysis

#### 2.5.1. Correlation Analysis of the Trace Elements

An analysis of the correlation between the elements determined was conducted using the raw HR-ICP-MS analysis of 34 elements from digested filters during each sampling period. To determine the most appropriate trace elements associated with emissions from the well site and to discriminate emissions from intervening sources, a logic tree model was established. While there may be elements that are uniquely associated with the well pad emissions, if the concentration of those elements is not sufficient to allow measurement of them, they cannot be considered. For exclusion criterion 1 (detectable mass), therefore, elements were excluded when total concentration on the filter did not rise above the quantification limit at any of the downwind locations.

Flow-corrected measurements were calculated by dividing the total mass for each element by total volume measured with the dry gas meter (ng/m^3^ = ng trace element/m^3^ of air flow) (Table S2). Using these flow-corrected measurements, it was assumed that the downwind concentrations coming from the well pad would decrease with distance. It is possible under turbulent wind conditions that some points downwind may experience momentary increases in concentration from an upwind source. Because the data were collected over a period of several days, we considered that a constant or increasing trend at all downwind sites was unlikely if the material emanated from the well pad. Likewise, it was assumed that precipitation would affect concentrations equally at every site since the sites were located in a very close proximity to each other. All other meteorological parameters were held constant. Trace element concentrations were fit to a simple power function relationship (y = cxb) rather than try to fit a more complex Gaussian profile to reduce the potential of false negatives. Using the mass/volumetric concentrations, exclusion of any of the remaining elements not excluded by having an increasing trend were made by establishing whether or not the trace elements were decreasing as a power function with distance, as highlighted in Figure S3. To calculate the trendline, the distance used for the well site was 0.1 km, Site 1 was 0.92 km, Site 2 was 2.30 km, and Site 3 was 7.16 km.

**Figure 1:**
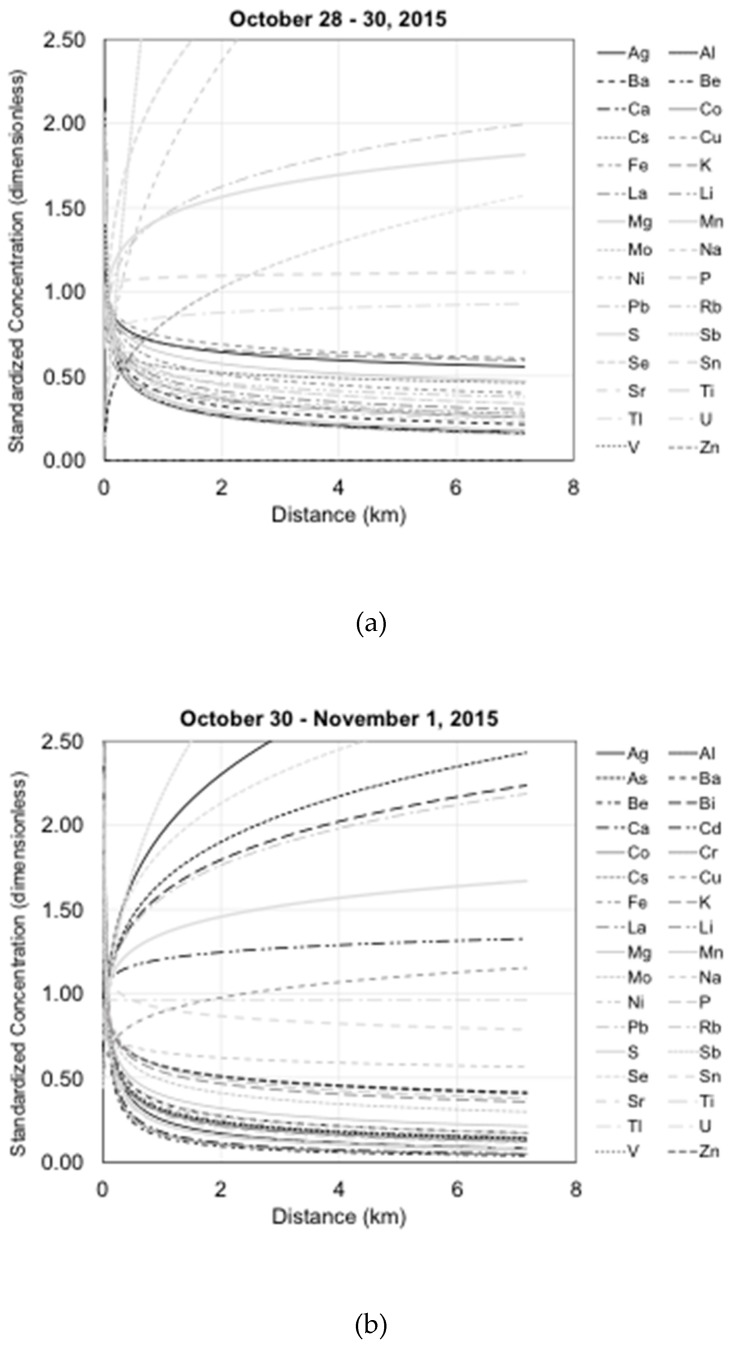

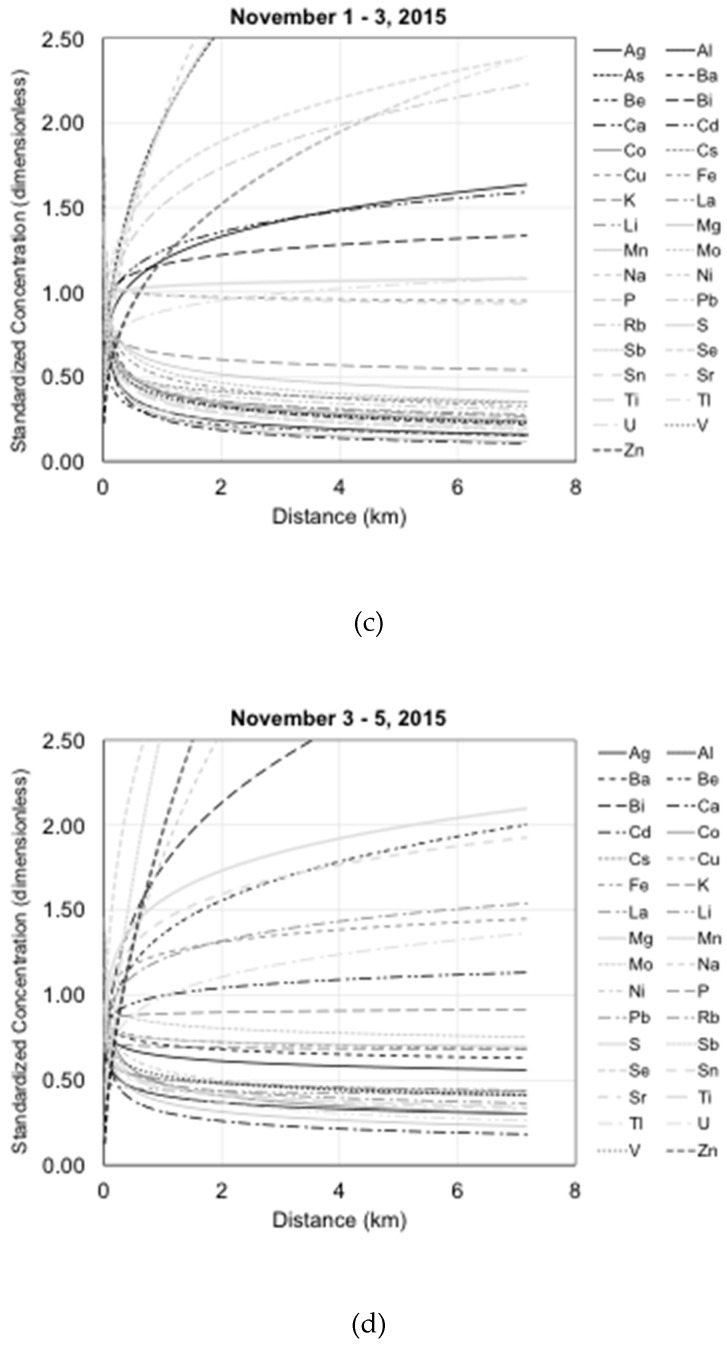

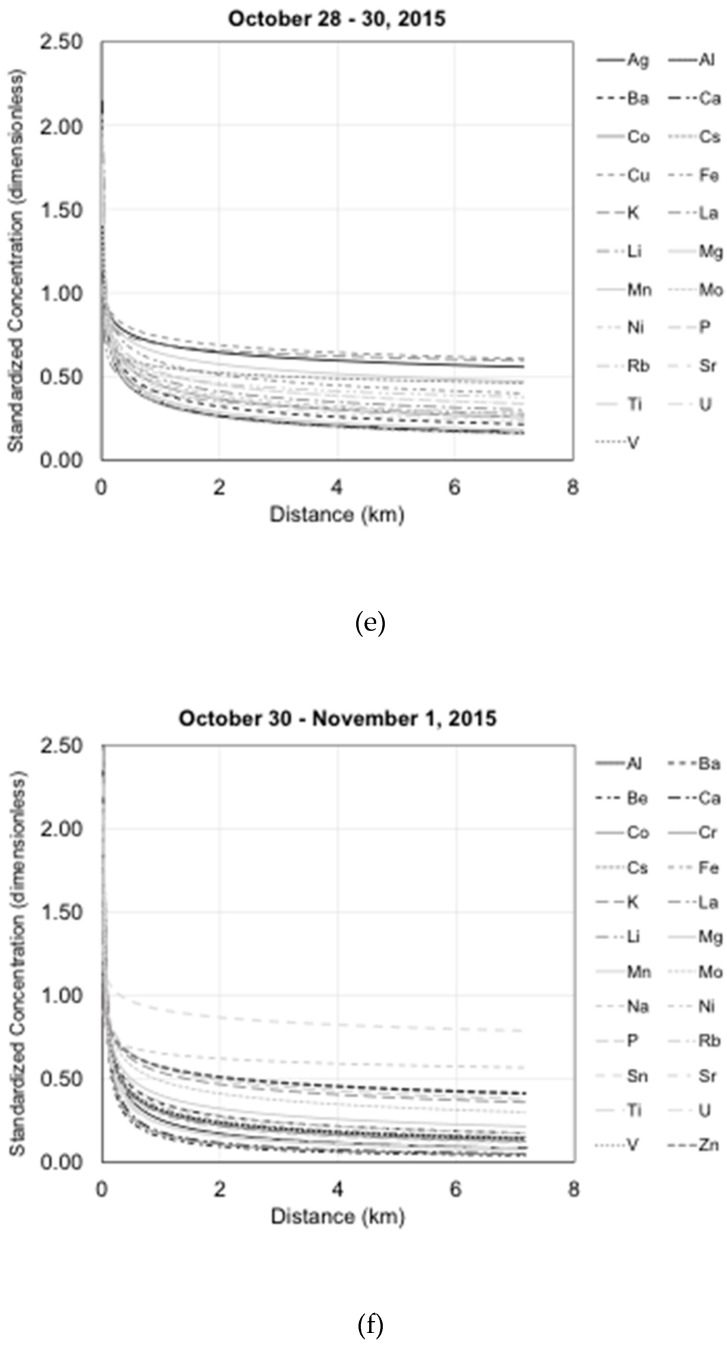

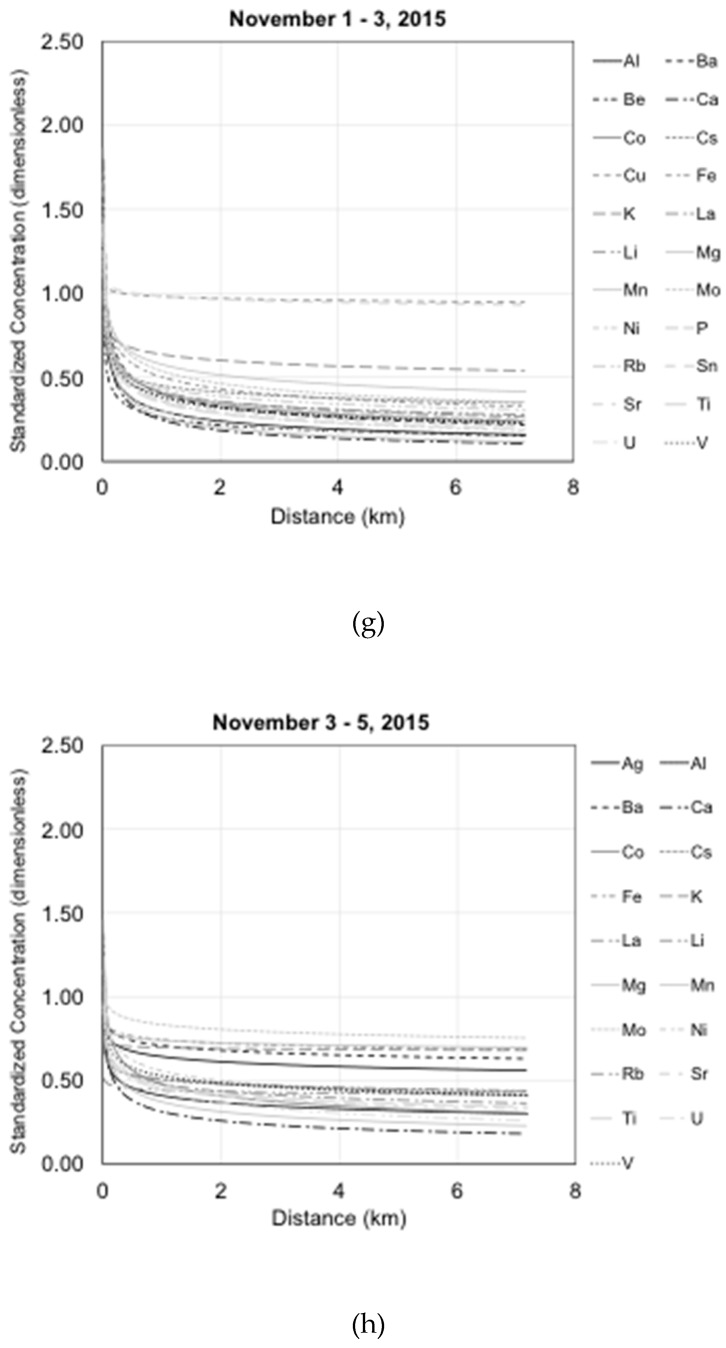
(**a**–**d**) Standardized concentration trends distinguishing which elements increase with distance (exclusion criteria 2); (**e**–**h**) standardized concentration trends distinguishing consistent diminution of concentration with distance (exclusion criteria 3).

As determined by a positive value of the exponent in the fitted power equation, those elements that increased with distance from the emission source at the well pad were assumed to likely indicate emissions arising from intervening sources and were thus not considered under exclusion criterion 2 (implausible increase in concentration with distance) (Figure 1a–d). For those elements remaining after exclusion criteria 2, a coefficient of determination (r^2^ > 0.6) for the power trendline was used to decide whether the decreasing concentrations were a consistent trend with distance downwind from the well site. It was assumed that the introduction of other sources of the tracers at intervening distances downwind would result in the increases in concentration and diminution of the correlation coefficient. It is, of course, still possible that the actual dispersion of the elemental tracers is not following the power function relationship exactly. However, this was still considered a conservative assumption for exclusion. Those elements with r^2^ < 0.6 were therefore excluded under exclusion criterion 3 (inconsistent diminution of concentration with distance) (Figure 1e–h).

The next assumption was that a true tracer of emissions would likely be proportional to other elements. To further reduce the possibility of confounding sources, strong proportional fit between elements was concluded if the r^2^ value between elements was greater than 0.6 and the acceptable slope of the comparison of the selected element to three or more other elements during the sampling period was nominally zero but at least between values of plus and minus one. This served as exclusion criteria 4 (inconsistent proportionality over distance between elements with fewer than three other trace elements). We assumed that the wind would need to be blowing at least 1 mile per hour in the direction of the sampling sites at least five percent of the time in order to detect trace elements (Table S3). Timeframes when wind patterns did not meet these criteria were excluded in criteria 5. Finally, to be most conservative, all elements where a strong proportional fit of three or more elements was not established both within and between sampling periods were excluded in criteria 6. 

#### 2.5.2. PM2.5 Measurement

Simultaneous direct-reading PM2.5 mass measurements (mg/m3) were performed at each site using TSI DustTrak™ II 8530 Aerosol Monitors during the sampling periods. Measurements were taken every minute at all sites except for the well site which was taken every two minutes. DustTrak™ monitors were situated beside other monitoring devices downwind of UNGD operations. Raw data from these experiments were saved in a Microsoft Excel^®^ document.

These PM2.5 mass measurements were corrected against a factory calibrated DustTrak device, and the corrected DustTrak values were then averaged daily and compared to the elemental data.

We selected the October 30–November 1, 2015 sampling period because it had the most consistent dust generation from the well site along with appropriate windspeed and direction. Distance was inserted into the elemental power trendline calculation for the October 30–November 1, 2015 sampling period using a distance of 0.1 km for the well site. This established the elemental value to daily DustTrak average by distance. We then compared these values using by Microsoft Excel® power trendline calculation. This was repeated for all elements deemed significant during the October 30–November 1, 2015 sampling period to determine whether we could use the DustTrak alone to follow the plume profile.

## 3. Results 

### Elemental Analysis

The 34 trace elements initially considered for inclusion in the analysis for each sampling period are listed in Table S1. For the October 28–30, 2015 sampling period, As, Bi, Cd, and Cr (n = 4) did not exceed the detection limit; Be, Na, Pb, S, Sb, Se, Sn, Tl, and Zn were excluded because their concentration did not decrease overall with distance (n = 9); Cs, Cu, K, Li, Ni, and Rb (n = 6) did not have a strong association for proportional concentration decrease in distance; Ag, Al, Ba, Ca, Co, Fe, La, Mn, P, Sr, Ti, U, and V (n = 13) did not demonstrate a strong proportional fit with (r^2^ > 0.6, slope 1) to at least three other elements during the sampling period; and Mo (n = 1) was excluded due to lack of proportionality to at least three other elements across other sampling periods. Wind pattern data demonstrated that the wind was blowing greater than 1 mile per hour (mph) from the directions of interest (SE, SSE, S, S, SSW, SW) for approximately 42% of the overall sampling time. Results of the wind analysis for Oct. 28–30 are listed in Table S3. Mg, which demonstrated proportionality to Ba (r^2^ = 0.91, slope = 0.008), Co (r^2^ = 0.66, slope = 0.0001), Sr (r^2^ = 0.79, slope = 0.002), U (r^2^ = 0.87, slope = 0.00001), and V (r^2^ = 0.68, slope = 0.001), was the only remaining element to match the selection criteria.

For the October 30–November 1 2015 sampling period, all elements met the detection limit. Ag, As, Bi, Cd, Cu, Pb, S, Sb, Se, Tl (n = 10) were excluded because their concentration did not decrease overall with distance, while Be, Li, Na, Sn, Zn (n = 5) did not have a strong association for proportional concentration decrease in distance. Al, Ba, Ca, Co, Cs, K, Mn, Mo, Ni, P, Rb, Sr, V (n = 13) did not demonstrate a strong proportional fit with (r^2^ > 0.6, slope 1) to at least three other elements during the sampling period. Wind pattern data demonstrated that the wind was blowing greater than 1 mile per hour (mph) from the directions of interest (SE, SSE, S, S, SSW, SW) for approximately 38% of the overall sampling time. Cr, Fe, La, Ti, U (n = 5) were excluded due to lack of proportionality to at least three other elements across other sampling periods. Similarly with the previous sampling period, Mg was the only remaining element to meet all of the selection criteria, and demonstrated proportionality to Ba (r^2^ = 0.72, slope = 0.032), Co (r^2^ = 0.97, slope = 0.0001), Sr (r^2^ = 0.99, slope = 0.003), U (r^2^ = 0.72, slope = 0.00001), V (r^2^ = 0.94, slope = 0.0008), in addition to Cs (r^2^ = 0.97, slope = 0.0001), Fe (r^2^ = 0.92, slope = 0.372), La (r^2^ = 0.94, slope = 0.0002), Mn (r^2^ = 0.75, slope = 0.014), Ni (r^2^ = 0.87, slope = 0.0001), Rb (r^2^ = 0.87, slope = 0.0001), and Ti (r^2^ = 0.76, slope = 0.016). 

During the November 1–3, 2015 sampling period, Cr (n = 1) was the only element excluded for not meeting the detection limit. Additionally, Ag, As, Bi, Cd, Na, Pb, S, Sb, Se, Tl, and Zn (n = 11) were excluded because their concentration did not decrease overall with distance, and Al, Be, Ca, Co, Cs, Cu, K, La, Li, Mn, P, Rb, Sn, Sr, Ti, U, and V (n = 17) did not have a strong association for proportional concentration decrease in distance. Ba, Fe, Mo and Ni (n = 4) were excluded for not having a strong proportional fit to at least three other elements. This left Mg (n = 1) as the only remaining element that demonstrated a strong proportional fit (r^2^ > 0.6, slope 1) to at least three other elements including Ba (r^2^ = 0.72, slope = 0.032), which is consistent to the October 28–30 and October 30–November 1 sampling periods, Fe (r^2^ = 0.92, slope = 0.372), and Ni (r^2^ = 0.87, slope = 0.001). Wind patterns indicate that the wind was blowing in the directions of interest for 11% of the total sample period.

The November 3–5, 2015 sampling period demonstrated the weakest correlations of any sampling period. As and Cr (n = 2) were excluded for concentrations not meeting the detection limits, followed by exclusion of Be, Bi, Cd, Cu, Na, P, Pb, S, Sb, Se, Sn, Tl, and Zn (n = 13) for concentration not decreasing overall with distance. All remaining elements Ag, Al, Ba, Ca, Co, Cs, Fe, K, La, Li, Mg, Mn, Mo, Ni, Rb, Sr, Ti, U, V (n = 19) were excluded for lack of strong association for proportional concentration decrease in distance prior to even considering the role of wind patterns, which only met the criteria of interest approximately 4.5% of the time.

Results of the trace elemental analysis are summarized in Table 1. Mg concentrations were consistently proportional to other elements across all three of our included sampling time periods (October 28–30, October 30–November 1, November 1–3, 2015). During the two sampling periods (October 28–30, October 30–November 1, 2015), the wind blew towards our samplers greater than 35% of the time (Table 2), and Mg was consistently proportional to (Ba, Co, Sr, U, and V) at each sampling site.

## 4. Discussion

In this hypothesis generation study, we collected PM2.5 samples at three points downwind of a UNGD well pad in West Virginia during the stimulation process and used HR-ICP-MS to analyze trace elemental content. We sought to determine 1) what trace elements unique to the UNGD well pad could be identified at distances downwind, and 2) whether these elements corresponded to PM2.5 measurements. With this information, we developed an experimental source identification model for the wind patterns and topography of the surrounding area to test our hypotheses.

Limited information about the source of Mg is available for UNGD. Mg is a major element in the earth’s crust and is present in a variety of minerals, especially mafic minerals [37]. It is known to derive from marine sources such as sea salt [38] and industrial emissions (such as coal combustion and ceramics manufacturing) [35] as well as from diesel emissions [39]. In 2011, Adams found that application of hydrofracturing fluid to an experimental test forest in WV led to elevated soil Mg levels. Similarly, Ca, Al, Zn, and Mn were also elevated in soil treated with hydrofracturing fluids. However, these elements were not determined to demonstrate proportional decay from the well site [40]. Mg is known component of hydraulic fracturing fluid and used as a gel breaker (MgO, MgO2) in the process [41]. It is not known how Mg could be aerosolized during the fracturing process that would lead to downwind airshed contamination. Alternatively, could be emitted as a component of diesel particulate matter, likely from the lube oil [39,42,43].

While Mg may not serve as a generalizable trace element, this source identification model could be used to identify localized trace elements in different shale plays and for different point sources. It should be noted that although air emissions could be detected at our furthest sampling point at 7 km, the highest emission levels were detected on site at the gas well. It appears that when the wind is blowing at speeds greater than 1 mph towards the sampling devices, more often, there is a greater number of proportional correlations.

Our model for identifying tracers is a more sensitive way to distinguish a PM2.5 derived source from background for the particular topography, meteorology, shale play at depth, and UNGD process unique to the location and timeframe being sampled. While these results should not be immediately extrapolated to other sources, we hypothesize that this method of elemental identification can be used to characterize the distance that PM2.5 air emissions travel from any given point source. Further studies using this model could assist in developing an exposure matrix for epidemiological studies assessing off-site exposures to PM2.5.

The trace elements from the UNGD well pad we analyzed decline after 1 km, consistent with the 2012 study by McKenzie et al. [26]. Other papers using inverse-distance squared metrics as a surrogate for exposure have identified adverse health outcomes for people living within 10 miles of a UNGD well [24,27]. This is twice as far as our furthest sampling device at 7 km, where we detected significantly diminished levels. However, one major limitation of our model design is that it eliminates confounding sources not generated directly on the UNGD well pad. Our model eliminates other sources of air emissions that may be directly UNGD-related and namely eliminates the contribution of UNGD-related truck traffic, which we believe to be a significant source of air emissions considering that 7000 to 11,000 one-way truck trips are required for the development of a single UNGD well [44,45]. These sources are mobile and stretched out across great distances and are not directly monitored or regulated. Waste from UNGD wells in Pennsylvania can be transported anywhere between 106 and 237 km from a well pad to the disposal site, and local roads receive the greatest amount of truck traffic [46]. Because such significant diesel truck traffic is required for UNGD, future studies using trace analysis or other methods that assist in identifying the extent of diesel truck traffic emissions are needed to understand why we may be seeing increased adverse health effects out as far as 10 km from UNGD.

There were other limitations of our study. Disturbances in the flow sampling at Site 0 resulted in suboptimal vacuum pressure as described in Table S1. We speculate that this may have caused the cyclone to not properly filter out larger particles. However, due to the high mass concentrations of materials, we do not believe it had significant effect on our analysis. Further, the stimulation process did not begin until October 29, which means that our first sampling period (October 28–30, 2015), included one day of background samples, which may have washed out source tracer to the stimulation process during the sample period, though this did not appear to affect our conclusions for other sampling periods.

Using exclusion criteria based on a coefficient of determination of r^2^ > 0.6 may be too conservative an approach. For the purposes of this study, this likely excluded a number of elements simply because there was not enough dust detected at 7 km out from the well due to the reduced concentration of PM2.5 at this sampling site. 

Future studies should work to confirm our initial findings at other wells in the Marcellus Shale region. Furthermore, these techniques could be applicable to other UNGD in the Barnett, Utica, and other reservoirs.

### Correlation of PM2.5 Measurements to Elemental Data

Regression analysis of elemental concentrations to PM2.5 measurements were not statistically significant, indicating that PM2.5 measurements do not adequately serve as a surrogate for trace elemental analysis. The following results were obtained from the October 30–November 1, 2015 comparison of PM2.5 measurements to elements: Mg (r^2^ = 0.278), Cr (r^2^ = 0.253), La (r^2^ = 0.444), Fe (r^2^ = 0.477), Ti (r^2^ = 0.352), and U (r^2^ = 0.355) (Figure S4).

## 5. Conclusions

Findings indicate that Mg is consistently proportional to multiple other elements at sampling sites 7 km downwind of the Morgantown UNGD well site, with a correlation of r^2^ > 0.6. These results provide strong evidence that Mg can be used as a tracer to detect off-site PM2.5 emissions generated during the stimulation process at the UNGD well site. 

## Figures and Tables

**Table 1:** Selection criteria for elements per sample period ^1^.

Exclusion Criteria					
	Sampling Period	Elements Excluded	Excluded N	Elements Included	Included N
**EXCLUSION 1: Concentration exceeded detection limit**					
	10/28/15 to 10/30/15	As, Bi, Cd, Cr	4	Ag, Al, Ba, Be, Ca, Co, Cs, Cu, Fe, K, La, Li, Mg, Mn, Mo, Na, Ni, P, Pb, Rb, S, Sb, Se, Sn, Sr, Ti, Tl, U, V, and Zn	30
	10/30/15 to 11/1/15	-	0	Ag, Al, As, Ba, Be, Bi, Ca, Cd, Co, Cr, Cs, Cu, Fe, K, La, Li, Mg, Mn, Mo, Na, Ni, P, Pb, Rb, S, Sb, Se, Sn, Sr, Ti, Tl, U, V, and Zn	34
	11/1/15 to 11/3/15	Cr	1	Ag, Al, As, Ba, Be, Bi, Ca, Cd, Co, Cs, Cu, Fe, K, La, Li, Mg, Mn, Mo, Na, Ni, P, Pb, Rb, S, Sb, Se, Sn, Sr, Ti, Tl, U, V, and Zn	33
	11/3/15 to 11/5/15	As, Cr	2	Ag, Al, Ba, Be, Bi, Ca, Cd, Co, Cs, Cu, Fe, K, La, Li, Mg, Mn, Mo, Na, Ni, P, Pb, Rb, S, Sb, Se, Sn, Sr, Ti, Tl, U, V, and Zn	32
**EXCLUSION 2: Concentration decreased overall with distance**					
	10/28/15 to 10/30/15	Be, Na, Pb, S, Sb, Se, Sn, Tl, Zn	9	Ag, Al, Ba, Ca, Co, Cs, Cu, Fe, K, La, Li, Mg, Mn, Mo, Ni, P, Rb, Sr, Ti, U, and V	21
	10/30/15 to 11/1/15	Ag, As, Bi, Cd, Cu, Pb, S, Sb, Se, Tl	10	Al, Ba, Be, Ca, Co, Cr, Cs, Fe, K, La, Li, Mg, Mn, Mo, Na, Ni, P, Rb, Sn, Sr, Ti, U, V, and Zn	24
	11/1/15 to 11/3/15	Ag, As, Bi, Cd, Na, Pb, S, Sb, Se, Tl, Zn	11	Al, Ba, Be, Ca, Co, Cs, Cu, Fe, K, La, Li, Mg, Mn, Mo, Ni, P, Rb, Sn, Sr, Ti, U, and V	22
	11/3/15 to 11/5/15	Be, Bi, Cd, Cu, Na, P, Pb, S, Sb, Se, Sn, Tl, Zn	13	Ag, Al, Ba, Ca, Co, Cs, Fe, K, La, Li, Mg, Mn, Mo, Ni, Rb, Sr, Ti, U, and V	19
**EXCLUSION 3: r^2^ values > 0.6**					
	10/28/15 to 10/30/15	Cs, Cu, K, Li, Ni, Rb	6	Ag, Al, Ba, Ca, Co, Fe, La, Mg, Mn, Mo, P, Sr, Ti, U, V	15
	10/30/15 to 11/1/15	Be, Li, Na, Sn, Zn	5	Al, Ba, Ca, Co, Cr, Cs, Fe, K, La, Mg, Mn, Mo, Ni, P, Rb, Sr, Ti, U, and V	19
	11/1/15 to 11/3/15	Al, Be, Ca, Co, Cs, Cu, K, La, Li, Mn, P, Rb, Sn, Sr, Ti, U, V	17	Ba, Fe, Mg, Mo, and Ni	5
	11/3/15 to 11/5/15	Ag, Al, Ba, Ca, Co, Cs, Fe, K, La, Li, Mg, Mn, Mo, Ni, Rb, Sr, Ti, U, V	19	-	0
**EXCLUSION 4: Strong proportional fit to at least 3 other elements per period (r^2^ > 0.6, slope approx. +/- 1)**					
	10/28/15 to 10/30/15	Ag, Al, Ba, Ca, Co, Fe, La, Mn, P, Sr, Ti, U, V	13	Mg and Mo	2
	10/30/15 to 11/1/15	Al, Ba, Ca, Co, Cs, K, Mn, Mo, Ni, P, Rb, Sr, V	13	Cr, Fe, La, Mg, Ti, and U	6
	11/1/15 to 11/3/15	Ba, Fe, Mo, Ni	4	Mg	1
	11/3/15 to 11/5/15	-	-	-	-
**EXCLUSION 5: Wind blowing > 1 mph in proper direction more than 5% of time**					
	10/28/15 to 10/30/15	-	0	Mg and Mo	2
	10/30/15 to 11/1/15	-	0	Cr, Fe, La, Mg, Ti, and U	6
	11/1/15 to 11/3/15	-	0	Mg	1
	11/3/15 to 11/5/15	excludes this sampling period	-	-	-
**EXCLUSION 6: Proportional to at least 3 other elements across all remaining sample periods**					
	10/28/15 to 10/30/15	Mo	1	Mg ^2^	1
	10/30/15 to 11/1/15	Cr, Fe, La, Ti, U	5	Mg ^3^	1
	11/1/15 to 11/3/15	-	0	Mg ^4^	-
	11/3/15 to 11/5/15	-	-	-	-

Note: ^1^ Elements analyzed (n = 34): Ag, Al, As, Ba, Be, Bi, Ca, Cd, Co, Cr, Cs, Cu, Fe, K, La, Li, Mg, Mn, Mo, Na, Ni, P, Pb, Rb, S, Sb, Se, Sn, Sr, Ti, Tl, U, V, and Zn; ^2^ Mg is proportional to Ba (r^2^ = 0.91, slope = 0.008), Co (r^2^ = 0.66, slope = 0.0001), Sr (r^2^ = 0.79, slope = 0.002), U (r^2^ = 0.87, slope = 0.00001), and V (r^2^ = 0.68, slope = 0.001); ^3^ Mg is proportional to Ba (r^2^ = 0.72, slope = 0.032), Co (r^2^ = 0.97, slope = 0.0001), Sr (r^2^ = 0.99, slope = 0.003), U (r^2^ = 0.72, slope = 0.00001), V (r^2^ = 0.94, slope = 0.0008), Cs (r^2^ = 0.97, slope = 0.0001), Fe (r^2^ = 0.92, slope = 0.372), La (r^2^ = 0.94, slope = 0.0002), Mn (r^2^ = 0.75, slope = 0.014), Ni (r^2^ = 0.87, slope = 0.0001), Rb (r^2^ = 0.87, slope = 0.0001), and Ti (r^2^ = 0.76, slope = 0.016); ^4^ Mg is proportional to Ba (r^2^ = 0.72, slope = 0.032), Fe (r^2^ = 0.92, slope = 0.372), and Ni (r^2^ = 0.87, slope = 0.001).

**Table 2:** Magnesium concentration and windspeed.

			Total ^2^	Wind > 1 mph ^3^				Wind < 1 mph ^4^			
Sampling Period	Sampling Site	Mg (ng) ^1^	n	n	Mean	SD	% time	n	Mean	SD	% time
10/28–10/30	0 km	22.7	338	143	7.2	9.0	42.3%	82	16.4	19.9	24.3%
	1 km	7.6									
	2 km	4.2									
	7 km	5.7									
10/30–11/1	0 km	63.5	348	133	6.7	9.8	38.2%	133	26.6	30.4	38.2%
	1 km	14.5									
	2 km	5.9									
	7 km	6.6									
11/1–11/3	0 km	72.4	337	38	1.9	1.8	11.3%	176	35.2	38.3	52.2%
	1 km	6.9									
	2 km	14.2									
	7 km	16.8									
11/3–11/5	0 km	82.6	333	15	0.8	1.1	4.5%	145	29.0	33.4	43.5%
	1 km	24.7									
	2 km	12.6									
	7 km	31.2									

Note: ^1^ Flow-corrected concentration in ng/m^3^; ^2^ total number of wind events captured during sampling period travelling downwind; ^3^ mph = miles per hour; events measured at >1 mph travelling downwind towards sampling stations; ^4^ events measured at <1 mph travelling downwind towards sampling stations.

## Supplementary Materials

The following are available online at https://www.mdpi.com/1660-4601/17/6/1837/s1, Figure S1: Timeline of unconventional natural gas development at Marcellus Shale well site in Morgantown, West Virginia, Figure S2: Map of four air sampling sites by approximate 1 km, 2 km and 7 km distances from UNGD well site in Morgantown, West Virginia, Figure S3: Sample least squares fit test. Negative exponents determined whether concentration decreased by distance. Strength of fit was determined by r^2^ > 0.6, Figure S4: Correlation of PM2.5 measurements to elemental data, Table S1: HR-ICP-MS results from the October 28–November 5, 2015 sampling period, Table S2: Flow measurements for elemental sampling, and Table S3: Wind patterns during an 8 day hydraulic fracturing stimulation process from the October 28–November 5, 2015 sampling period in Morgantown, West Virginia.
