# Supplementary material for: Use of Tracer Elements for Estimating Community Exposure to Marcellus Shale Development Operations
Source: Int J Environ Res Public Health. 2020 Mar 12;17(6):1837. doi: 10.3390/ijerph17061837 (PMC7143288; doi:10.3390/ijerph17061837)
Supplement: Supplementary file 1 [file ijerph-17-01837-s001.pdf]

Title: Use of Tracer Elements for Estimating Community Exposure to Marcellus Shale Development Operations

Abbreviated title: Potential tracer elements for fracking exposure assessment

Authorship: Maya J. Nye[1], Travis L. Knuckles[1], Beizhan Z. Yan[2], James M. Ross[2], Matthew S. Varonka[3], William H. Orem[3], George D. Thurston[4], Alexandria R. Dzomba[1], Michael A. McCawley[1]*

[1] West Virginia University School of Public Health, Department of Occupational and Environmental Health Sciences, PO Box 9190, Morgantown, WV 26506-9190
[2] The Lamont-Doherty Earth Observatory at Columbia University, 61 Route 9W - PO Box 1000, Palisades, NY 10964-8000
[3] US Geological Survey, 956 National Center, Reston, VA 201924
[4] Program in Exposure Assessment and Human Health Effects at the Department of Environmental Medicine, New York University School of Medicine, 57 Old Forge Road, Tuxedo, NY 10987

*Corresponding Author: Michael McCawley, PhD
Department of Occupational and Environmental Health Sciences
West Virginia University School of Public Health
PO Box 9190
Morgantown, WV 26506-9190
Tel: 304-293-2145 (office)
Email: mamccawley@hsc.wvu.edu

SUPPORTING INFORMATION:
Number of Pages: 12
Number of Figures: 4
Number of Tables:3

SUPPORTING INFORMATION

Figure Legend

Figure S1

Timeline of unconventional natural gas development at Marcellus Shale well site in Morgantown, West Virginia.

Figure S2

Map of four air sampling sites by approximate 1 km, 2 km and 7 km distances from UNGD well site in Morgantown, West Virginia.

Figure S3

Sample least squares fit test. Negative exponents determined whether concentration decreased by distance. Strength of fit was determined by $r^2 > 0.6$.

Figure S4

Correlation of PM2.5 measurements to elemental data.

Table Legend

Table S1

HR-ICP-MS results from October 28 - November 5, 2015.[1]

[1] Flow corrected values are listed in ng.

Table S2

Flow measurements for elemental sampling.

[1] Volume measured in cubic meter.

Table S3

Wind patterns during an 8-day hydraulic fracturing stimulation process from October 28 to November 5, 2015 in Morgantown, West Virginia.[1] (Primary information of interest is bolded.)

[1] Source: Weather Underground, Daily Weather History for Morgantown, WV - Brooks green from [KWVMORGA25]. Retrieved 2/9/15 from https://www.wunderground.com/personal-weather-station/dashboard?ID=KWVMORGA25#history

[2] mph = miles per hour

[3] WS = wind speed

[4] North (N), North-Northeast (NNE), Northeast (NE), East-Northeast (ENE), East (E), East-Southeast (ESE), Southeast (SE), South-Southeast (SSE), South (S), South-Southwest (SSW), Southwest (SW), West-Southwest (WSW), West (W), West-Northwest (WNW), Northwest (NW), North-Northwest (NNW)

[5] Percentage of direction by sampling timeframe

[6] Events of interest include direction from which the wind moves towards sampling stations (SE, SSE, S, SSW, SW) at > 1 mph

[7] Percentage of total wind direction across all sample periods

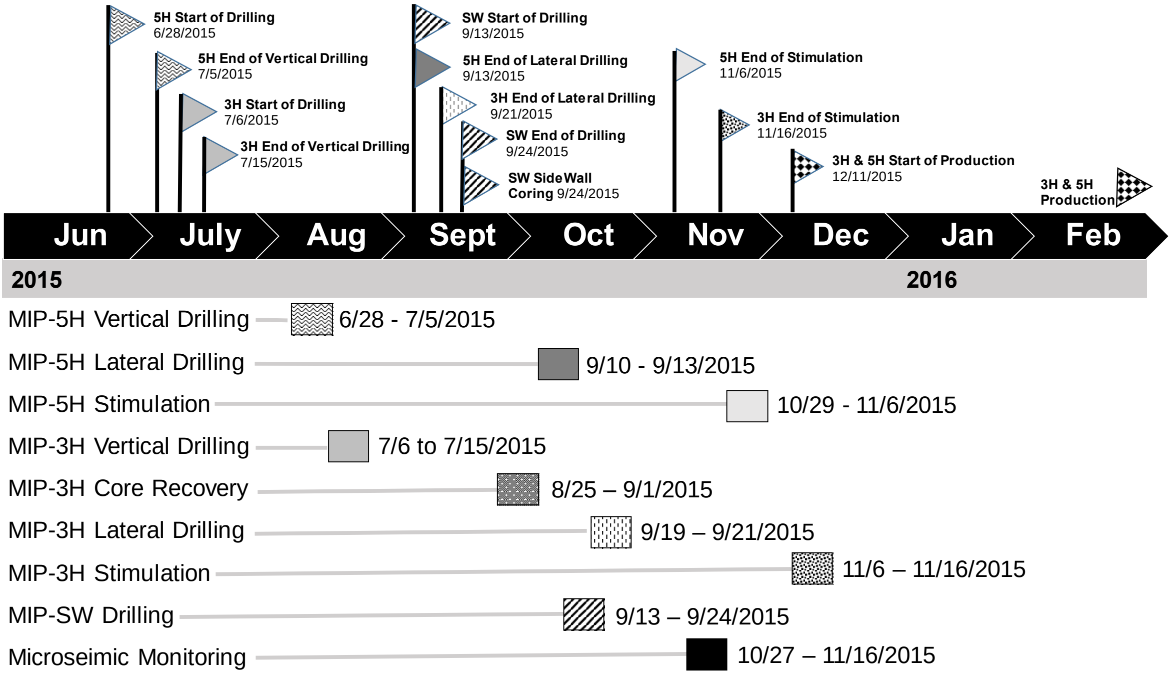

Figure S1. – Timeline of unconventional natural gas development at Marcellus Shale well site in Morgantown, WV.

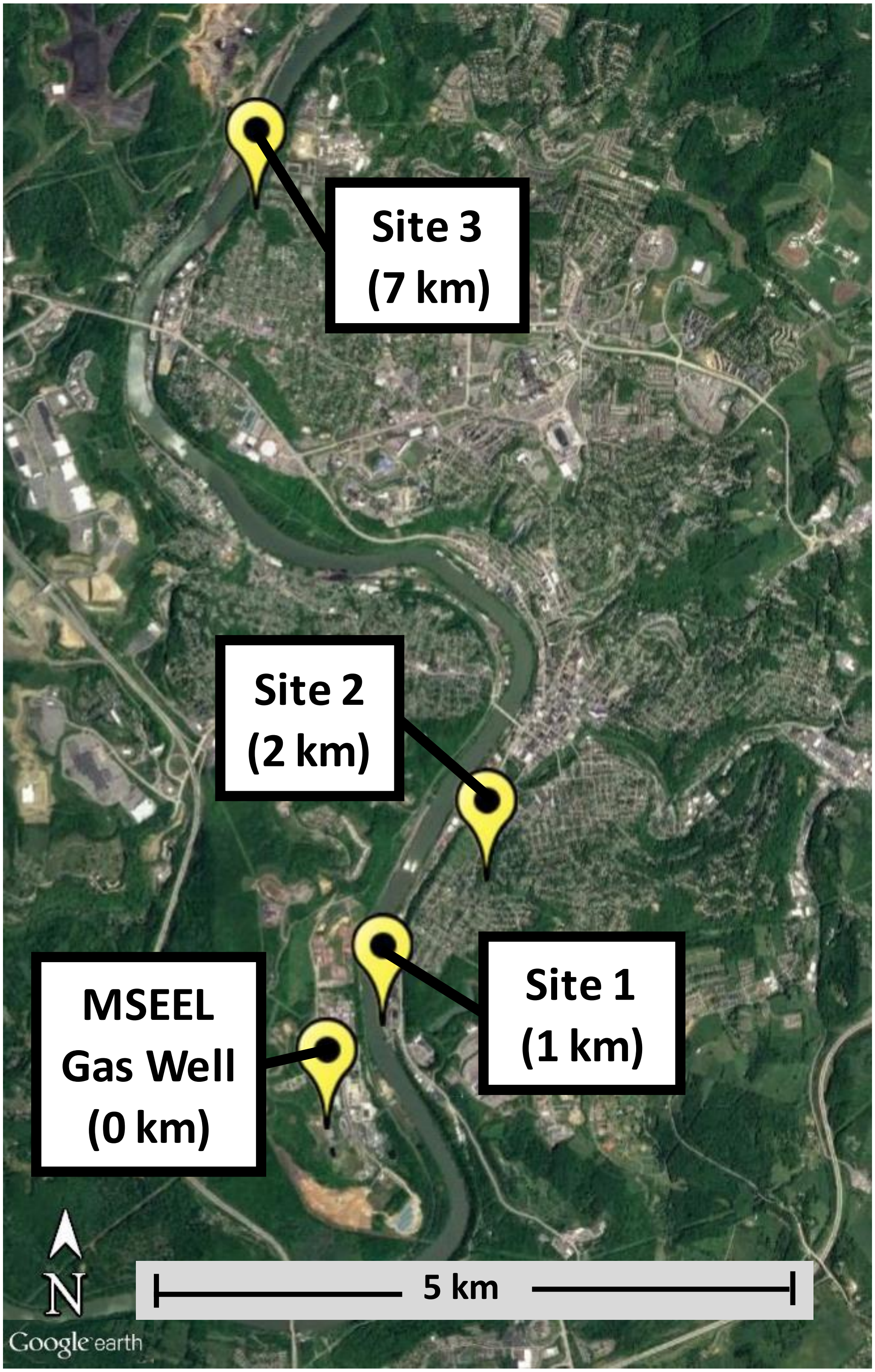

Figure S2. Map of four air sampling sites by approximate 1 km, 2 km and 7 km distances from UNGD well site in Morgantown, WV.

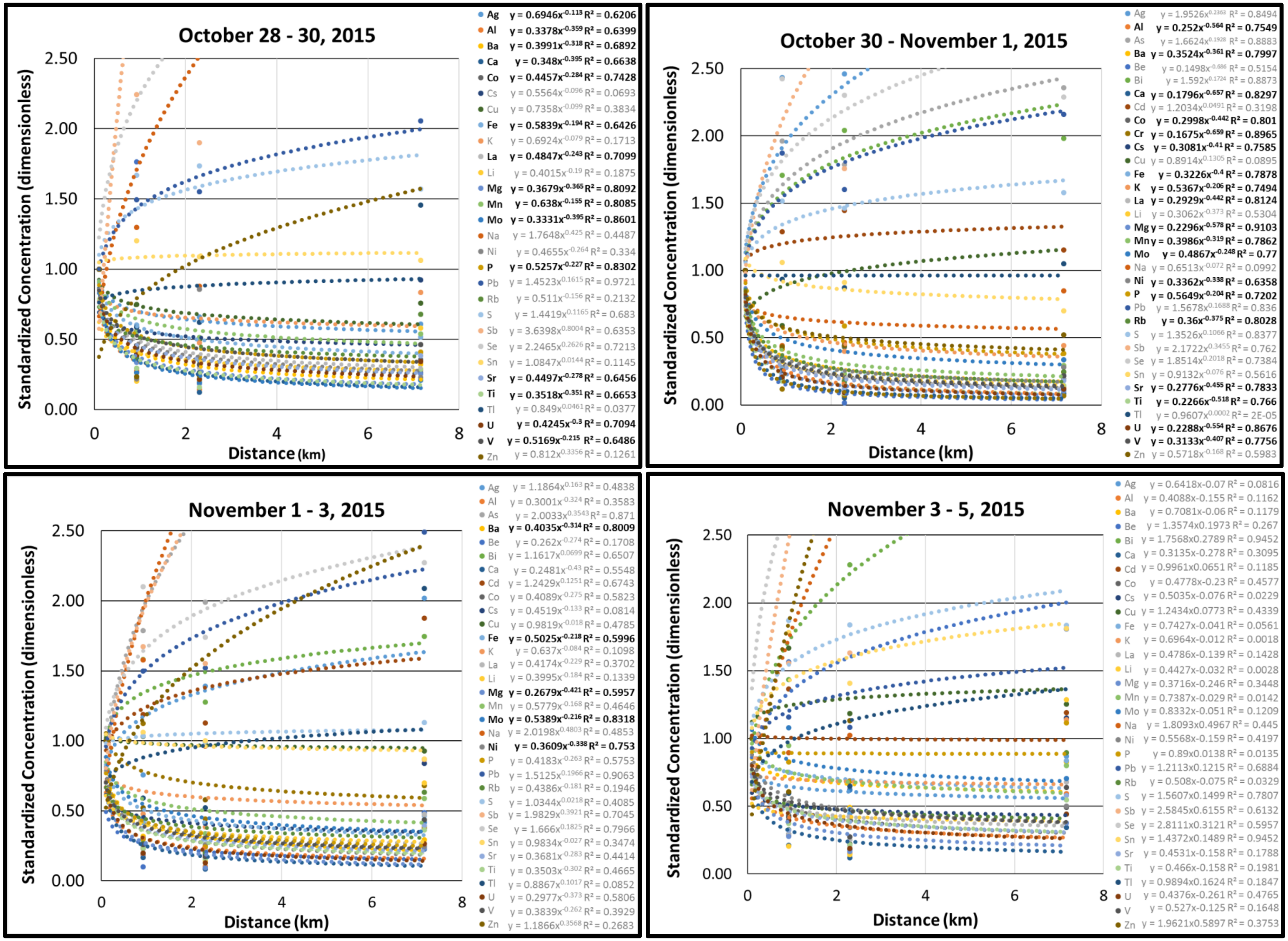

October 28 - 30, 2015
Standardized Concentration (dimensionless)
Distance (km)
Ag $y = 0.6946x^{-0.113}$ $R^2 = 0.6206$
Al $y = 0.3378x^{-0.359}$ $R^2 = 0.6399$
Ba $y = 0.3991x^{-0.318}$ $R^2 = 0.6892$
Ca $y = 0.348x^{-0.395}$ $R^2 = 0.6638$
Co $y = 0.4457x^{-0.284}$ $R^2 = 0.7428$
Cs $y = 0.5564x^{-0.096}$ $R^2 = 0.0693$
Cu $y = 0.7358x^{-0.099}$ $R^2 = 0.3834$
Fe $y = 0.5839x^{-0.194}$ $R^2 = 0.6426$
K $y = 0.6924x^{-0.079}$ $R^2 = 0.1713$
La $y = 0.4847x^{-0.243}$ $R^2 = 0.7099$
Li $y = 0.4015x^{-0.19}$ $R^2 = 0.1875$
Mg $y = 0.3679x^{-0.365}$ $R^2 = 0.8092$
Mn $y = 0.638x^{-0.155}$ $R^2 = 0.8085$
Mo $y = 0.3331x^{-0.395}$ $R^2 = 0.8601$
Na $y = 1.7648x^{0.425}$ $R^2 = 0.4487$
Ni $y = 0.4655x^{-0.264}$ $R^2 = 0.334$
P $y = 0.5257x^{-0.227}$ $R^2 = 0.8302$
Pb $y = 1.4523x^{0.1615}$ $R^2 = 0.9721$
Rb $y = 0.511x^{-0.156}$ $R^2 = 0.2132$
S $y = 1.4419x^{0.1165}$ $R^2 = 0.683$
Sb $y = 3.6398x^{0.8004}$ $R^2 = 0.6353$
Se $y = 2.2465x^{0.2626}$ $R^2 = 0.7213$
Sn $y = 1.0847x^{0.0144}$ $R^2 = 0.1145$
Sr $y = 0.4497x^{-0.278}$ $R^2 = 0.6456$
Ti $y = 0.3518x^{-0.351}$ $R^2 = 0.6653$
Tl $y = 0.849x^{0.0461}$ $R^2 = 0.0377$
U $y = 0.4245x^{-0.3}$ $R^2 = 0.7094$
V $y = 0.5169x^{-0.215}$ $R^2 = 0.6486$
Zn $y = 0.812x^{0.3356}$ $R^2 = 0.1261$
October 30 - November 1, 2015
Standardized Concentration (dimensionless)
Distance (km)
Ag $y = 1.9526x^{0.2363}$ $R^2 = 0.8494$
Al $y = 0.252x^{-0.564}$ $R^2 = 0.7549$
As $y = 1.6624x^{0.1928}$ $R^2 = 0.8883$
Ba $y = 0.3524x^{-0.361}$ $R^2 = 0.7997$
Be $y = 0.1498x^{-0.686}$ $R^2 = 0.5154$
Bi $y = 1.592x^{0.1724}$ $R^2 = 0.8873$
Ca $y = 0.1796x^{-0.657}$ $R^2 = 0.8297$
Cd $y = 1.2034x^{0.0491}$ $R^2 = 0.3198$
Co $y = 0.2998x^{-0.442}$ $R^2 = 0.801$
Cr $y = 0.1675x^{-0.659}$ $R^2 = 0.8965$
Cs $y = 0.3081x^{-0.41}$ $R^2 = 0.7585$
Cu $y = 0.8914x^{0.1305}$ $R^2 = 0.0895$
Fe $y = 0.3226x^{-0.4}$ $R^2 = 0.7878$
K $y = 0.5367x^{-0.206}$ $R^2 = 0.7494$
La $y = 0.2929x^{-0.442}$ $R^2 = 0.8124$
Li $y = 0.3062x^{-0.373}$ $R^2 = 0.5304$
Mg $y = 0.2296x^{-0.578}$ $R^2 = 0.9103$
Mn $y = 0.3986x^{-0.319}$ $R^2 = 0.7862$
Mo $y = 0.4867x^{-0.248}$ $R^2 = 0.77$
Na $y = 0.6513x^{-0.072}$ $R^2 = 0.0992$
Ni $y = 0.3362x^{-0.338}$ $R^2 = 0.6358$
P $y = 0.5649x^{-0.204}$ $R^2 = 0.7202$
Pb $y = 1.5678x^{0.1688}$ $R^2 = 0.836$
Rb $y = 0.36x^{-0.375}$ $R^2 = 0.8028$
S $y = 1.3526x^{0.1066}$ $R^2 = 0.8377$
Sb $y = 2.1722x^{0.3455}$ $R^2 = 0.762$
Se $y = 1.8514x^{0.2018}$ $R^2 = 0.7384$
Sn $y = 0.9132x^{-0.076}$ $R^2 = 0.5616$
Sr $y = 0.2776x^{-0.455}$ $R^2 = 0.7833$
Ti $y = 0.2266x^{-0.518}$ $R^2 = 0.766$
Tl $y = 0.9607x^{0.0002}$ $R^2 = 2E-05$
U $y = 0.2288x^{-0.554}$ $R^2 = 0.8676$
V $y = 0.3133x^{-0.407}$ $R^2 = 0.7756$
Zn $y = 0.5718x^{-0.168}$ $R^2 = 0.5983$
November 1 - 3, 2015
Standardized Concentration (dimensionless)
Distance (km)
Ag $y = 1.1864x^{0.163}$ $R^2 = 0.4838$
Al $y = 0.3001x^{-0.324}$ $R^2 = 0.3583$
As $y = 2.0033x^{0.3543}$ $R^2 = 0.871$
Ba $y = 0.4035x^{-0.314}$ $R^2 = 0.8009$
Be $y = 0.262x^{-0.274}$ $R^2 = 0.1708$
Bi $y = 1.1617x^{0.0699}$ $R^2 = 0.6507$
Ca $y = 0.2481x^{-0.43}$ $R^2 = 0.5548$
Cd $y = 1.2429x^{0.1251}$ $R^2 = 0.6743$
Co $y = 0.4089x^{-0.275}$ $R^2 = 0.5823$
Cs $y = 0.4519x^{-0.133}$ $R^2 = 0.0814$
Cu $y = 0.9819x^{-0.018}$ $R^2 = 0.4785$
Fe $y = 0.5025x^{-0.218}$ $R^2 = 0.5996$
K $y = 0.637x^{-0.084}$ $R^2 = 0.1098$
La $y = 0.4174x^{-0.229}$ $R^2 = 0.3702$
Li $y = 0.3995x^{-0.184}$ $R^2 = 0.1339$
Mg $y = 0.2679x^{-0.421}$ $R^2 = 0.5957$
Mn $y = 0.5779x^{-0.168}$ $R^2 = 0.4646$
Mo $y = 0.5389x^{-0.216}$ $R^2 = 0.8318$
Na $y = 2.0198x^{0.4803}$ $R^2 = 0.4853$
Ni $y = 0.3609x^{-0.338}$ $R^2 = 0.753$
P $y = 0.4183x^{-0.263}$ $R^2 = 0.5753$
Pb $y = 1.5125x^{0.1966}$ $R^2 = 0.9063$
Rb $y = 0.4386x^{-0.181}$ $R^2 = 0.1946$
S $y = 1.0344x^{0.0218}$ $R^2 = 0.4085$
Sb $y = 1.9829x^{0.3921}$ $R^2 = 0.7045$
Se $y = 1.666x^{0.1825}$ $R^2 = 0.7966$
Sn $y = 0.9834x^{-0.027}$ $R^2 = 0.3474$
Sr $y = 0.3681x^{-0.283}$ $R^2 = 0.4414$
Ti $y = 0.3503x^{-0.302}$ $R^2 = 0.4665$
Tl $y = 0.8867x^{0.1017}$ $R^2 = 0.0852$
U $y = 0.2977x^{-0.373}$ $R^2 = 0.5806$
V $y = 0.3839x^{-0.262}$ $R^2 = 0.3929$
Zn $y = 1.1866x^{0.3568}$ $R^2 = 0.2683$
November 3 - 5, 2015
Standardized Concentration (dimensionless)
Distance (km)
Ag y = 0.6418x-0.07 $R^2 = 0.0816$
Al y = 0.4088x-0.155 $R^2 = 0.1162$
Ba y = 0.7081x-0.06 $R^2 = 0.1179$
Be y = 1.3574x0.1973 $R^2 = 0.267$
Bi y = 1.7568x0.2789 $R^2 = 0.9452$
Ca y = 0.3135x-0.278 $R^2 = 0.3095$
Cd y = 0.9961x0.0651 $R^2 = 0.1185$
Co y = 0.4778x-0.23 $R^2 = 0.4577$
Cs y = 0.5035x-0.076 $R^2 = 0.0229$
Cu y = 1.2434x0.0773 $R^2 = 0.4339$
Fe y = 0.7427x-0.041 $R^2 = 0.0561$
K y = 0.6964x-0.012 $R^2 = 0.0018$
La y = 0.4786x-0.139 $R^2 = 0.1428$
Li y = 0.4427x-0.032 $R^2 = 0.0028$
Mg y = 0.3716x-0.246 $R^2 = 0.3448$
Mn y = 0.7387x-0.029 $R^2 = 0.0142$
Mo y = 0.8332x-0.051 $R^2 = 0.1209$
Na y = 1.8093x0.4967 $R^2 = 0.445$
Ni y = 0.5568x-0.159 $R^2 = 0.4197$
P y = 0.89x0.0138 $R^2 = 0.0135$
Pb y = 1.2113x0.1215 $R^2 = 0.6884$
Rb y = 0.508x-0.075 $R^2 = 0.0329$
S y = 1.5607x0.1499 $R^2 = 0.7807$
Sb y = 2.5845x0.6155 $R^2 = 0.6132$
Se y = 2.8111x0.3121 $R^2 = 0.5957$
Sn y = 1.4372x0.1489 $R^2 = 0.9452$
Sr y = 0.4531x-0.158 $R^2 = 0.1788$
Ti y = 0.466x-0.158 $R^2 = 0.1981$
Tl y = 0.9894x0.1624 $R^2 = 0.1847$
U y = 0.4376x-0.261 $R^2 = 0.4765$
V y = 0.527x-0.125 $R^2 = 0.1648$
Zn y = 1.9621x0.5897 $R^2 = 0.3753$

Figure S3. Sample least squares fit test. Negative exponents determined whether concentration decreased by distance. Strength of fit was determined by $r^2 > 0.6$.

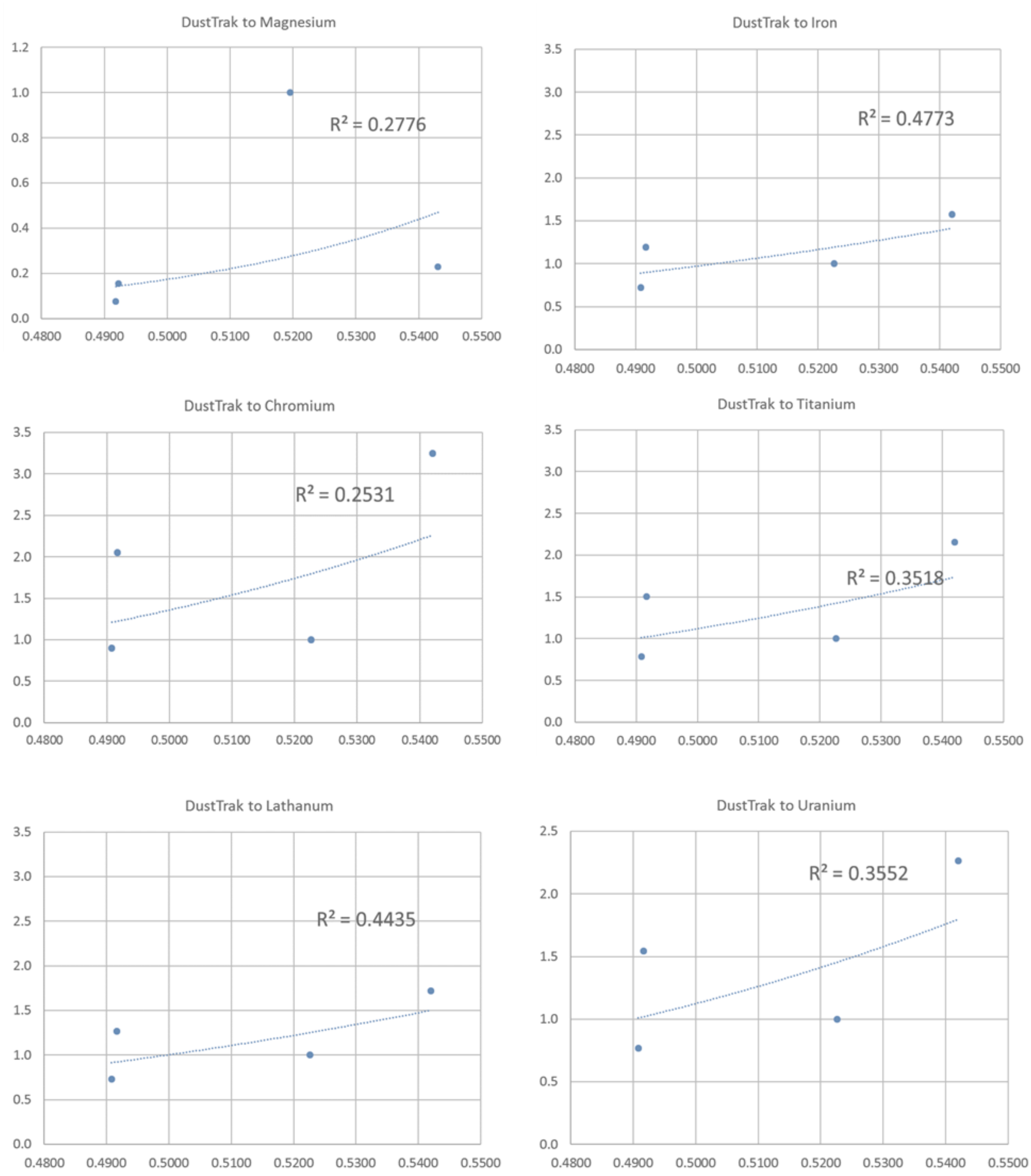

Figure S4. Correlation of PM2.5 measurements to elemental data.

Table S1. HR-ICP-MS results from October 28 - November 5, 2015.[1]

Elements (n=34)	UNGD Well Site (n=4)		Site 1 (n=4)		Site 2 (n=4)		Site 3 (n=4)	
	Mean	SD	Mean	SD	Mean	SD	Mean	SD
Ag	0.006	0.004	0.005	0.002	0.006	0.005	0.009	0.007
Al	213.010	96.247	60.352	32.453	23.663	14.068	81.342	73.235
As	0.218	0.151	0.462	0.274	0.586	0.426	1.192	0.938
Ba	14.365	7.923	6.060	4.705	4.327	3.408	6.595	6.246
Be	0.004	0.003	0.001	0.001	0.000	0.000	0.003	0.002
Bi	0.012	0.007	0.021	0.009	0.023	0.011	0.030	0.018
Ca	1002.611	642.775	211.310	134.240	90.653	74.281	251.299	251.104
Cd	0.088	0.073	0.123	0.102	0.085	0.045	0.125	0.089
Co	0.063	0.031	0.023	0.016	0.012	0.007	0.024	0.018
Cr	0.066	1.186	0.293	0.299	0.180	0.154	0.308	0.283
Cs	0.022	0.011	0.006	0.003	0.004	0.002	0.019	0.016
Cu	2.607	1.223	3.033	2.571	2.183	1.668	3.751	2.515
Fe	158.750	81.469	84.393	67.875	50.134	37.620	85.275	73.440
K	93.280	40.861	46.209	21.384	37.121	14.139	80.094	56.360
La	0.123	0.056	0.036	0.015	0.025	0.012	0.056	0.044
Li	1.109	0.714	0.248	0.165	0.168	0.102	0.977	0.904
Mg	60.296	26.270	15.271	7.055	7.378	3.649	15.075	11.852
Mn	3.959	1.743	2.612	2.220	1.343	0.593	2.339	1.694
Mo	0.339	0.099	0.202	0.174	0.141	0.076	0.149	0.099
Na	11.227	5.157	10.854	5.262	8.880	4.894	106.254	99.620
Ni	0.450	0.238	0.148	0.121	0.151	0.064	0.147	0.102
P	12.168	5.651	5.613	1.908	4.935	0.848	6.389	3.155
Pb	0.985	0.719	1.420	0.868	1.268	0.732	2.055	1.380
Rb	0.400	0.196	0.130	0.065	0.080	0.038	0.259	0.211
S	214.611	92.825	310.545	52.937	292.408	38.818	309.834	66.314
Sb	0.854	0.769	1.522	1.210	1.331	1.152	12.552	14.406
Se	0.162	0.086	0.462	0.147	0.413	0.121	0.414	0.163
Sn	0.426	0.204	0.558	0.306	0.453	0.218	0.467	0.298
Sr	1.977	1.110	0.595	0.378	0.340	0.225	0.839	0.754
Ti	14.113	6.431	4.023	2.878	2.165	1.462	5.180	4.394
Tl	0.007	0.003	0.005	0.002	0.005	0.001	0.015	0.010
U	0.023	0.011	0.007	0.005	0.003	0.002	0.007	0.006
V	0.359	0.159	0.125	0.075	0.075	0.039	0.165	0.123
Zn	12.405	4.523	6.280	4.236	8.225	7.252	167.940	214.497

[1] Flow corrected values are listed in ng/m^3.

Table S2. Flow measurements for elemental sampling.

Site Number	Start Date	TOTAL Volume (m^3)
0	10/28/15	9.491
1	10/28/15	50.224
2	10/28/15	47.586
3	10/28/15	48.011
0	10/30/15	8.614
1	10/30/15	47.587
2	10/30/15	50.633
3	10/30/15	49.726
0	11/1/15	5.422
1	11/1/15	48.487
2	11/1/15	46.658
3	11/1/15	49.731
0	11/3/15	4.286
1	11/3/15	52.344
2	11/3/15	48.324
3	11/3/15	48.588

[1] Volume measured in cubic meter.

Table S3. Wind patterns during an 8-day hydraulic fracturing stimulation process from October 28 to November 5, 2015 i Virginia.[1] (Primary information of interest is bolded.)

Speed (mph)[2]	WS[3] = < 1				WS = 1 to 2				WS = > 2 to 5			
Direction[4]	10/28 to 10/30	10/30 to 11/1	11/1 to 11/3	11/3 to 11/5	10/28 to 10/30	10/30 to 11/1	11/1 to 11/3	11/3 to 11/5	10/28 to 10/30	10/30 to 11/1	11/1 to 11/3	11/3 to 11/5
North	4	9	7	5	3	5	3	1	5	4	4	2
NNE	4	4	1	0	0	0	1	1	6	3	0	1
NE	1	1	0	0	0	0	0	0	2	2	1	1
ENE	1	0	1	2	0	0	0	0	1	1	1	0
East	0	2	1	0	0	0	1	0	0	0	0	0
ESE	0	1	0	0	1	0	0	0	0	1	0	0
**SE**	**1**	**3**	**0**	**2**	**0**	**1**	**2**	**0**	**2**	**1**	**3**	**0**
**SSE**	**19**	**29**	**96**	**86**	**4**	**3**	**1**	**0**	**24**	**23**	**7**	**2**
**South**	**50**	**78**	**34**	**30**	**5**	**3**	**0**	**2**	**32**	**32**	**5**	**1**
**SSW**	**7**	**8**	**41**	**13**	**5**	**1**	**1**	**2**	**6**	**6**	**1**	**1**
**SW**	**5**	**15**	**5**	**14**	**1**	**2**	**2**	**0**	**6**	**1**	**2**	**0**
WSW	3	15	9	27	0	0	1	4	2	0	2	8
West	4	6	18	1	0	0	0	0	3	0	0	0
WNW	0	4	37	2	0	0	0	0	0	0	0	1
NW	1	1	8	92	0	0	0	0	1	0	0	0
NNW	34	19	10	9	4	1	4	0	7	1	7	6
Events (n)	134	195	268	283	23	16	16	10	97	75	33	23
Events of interest (n)[6]	-	-	-	-	15	10	6	4	70	63	18	4

[1] Source: Weather Underground, Daily Weather History for Morgantown, WV - Brooks green from [KWVMORGA25]. Retr https://www.wunderground.com/personal-weather-station/dashboard?ID=KWVMORGA25#history

[2] mph = miles per hour

[3] WS = wind speed

[4] North (N), North-Northeast (NNE), Northeast (NE), East-Northeast (ENE), East (E), East-Southeast (ESE), Southeast (S South (S), South-Southwest (SSW), Southwest (SW), West-Southwest (WSW), West (W), West-Northwest (WNW), North (NNW)

[5] Percentage of direction by sampling timeframe

[6] Events of interest include direction from which the wind moves towards sampling stations (SE, SSE, S, SSW, SW) at > 1

[7] Percentage of total wind direction across all sample periods

Table S3 Continued

Speed (mph)[2]	ALL SPEEDS								GRAND TOTAL	
	**n**	**%[5]**	**n**	**%**	**n**	**%**	**n**	**%**	**n**	**%**
Direction[4]	10/28 to 10/30		10/30 to 11/1		11/1 to 11/3		11/3 to 11/5		10/28 to 11/5	
North	20	6%	18	5%	16	5%	8	2%	62	5%
NNE	12	4%	8	2%	2	1%	3	1%	25	2%
NE	5	1%	3	1%	1	0%	1	0%	10	1%
ENE	2	1%	1	0%	2	1%	2	1%	7	1%
East	1	0%	2	1%	2	1%	0	0%	5	0%
ESE	1	0%	2	1%	0	0%	0	0%	3	0%
**SE**	**7**	**2%**	**7**	**2%**	**7**	**2%**	**3**	**1%**	**24**	**2%**
**SSE**	**72**	**21%**	**81**	**23%**	**108**	**32%**	**91**	**27%**	**352**	**26%**
**South**	**108**	**32%**	**138**	**40%**	**43**	**13%**	**36**	**11%**	**325**	**24%**
**SSW**	**25**	**7%**	**20**	**6%**	**45**	**13%**	**16**	**5%**	**106**	**8%**
**SW**	**13**	**4%**	**20**	**6%**	**11**	**3%**	**14**	**4%**	**58**	**4%**
WSW	10	3%	15	4%	15	4%	47	14%	87	6%
West	8	2%	7	2%	18	5%	1	0%	34	3%
WNW	2	1%	4	1%	37	11%	3	1%	46	3%
NW	3	1%	1	0%	8	2%	92	28%	104	8%
NNW	49	14%	21	6%	22	7%	16	5%	108	8%
Events (n)	338		348		337		333		1356	
Events of interest (n)[6]	143	42%	133	38%	38	11%	15	4.5%	329	24%
